# Scale‐Invariant Waveguiding in Flatland

**DOI:** 10.1002/exp2.70108

**Published:** 2026-01-28

**Authors:** Zhixia Xu, Shuo Bao, Massimo Moccia, Giuseppe Castaldi, Tie Jun Cui, Vincenzo Galdi

**Affiliations:** ^1^ State Key Laboratory of Millimeter Waves Southeast University Nanjing China; ^2^ Fields & Waves Lab Department of Engineering University of Sannio Benevento Italy

**Keywords:** line waves, metasurfaces, polaritonics, surface waves, waveguiding

## Abstract

Electromagnetic metasurfaces with suitably designed spatial modulations of surface impedance can guide surface waves similarly to volumetric dielectric waveguides. As a result, the transverse distribution of the fundamental mode is usually nonuniform (peaked at the center), and its effective index is influenced by the electrical size of the central (core) region. Here, we introduce the concept of scale‐invariant surface waveguiding, extending the recent advancements in dielectric waveguides to flatland settings. By leveraging spatial symmetry and fine‐tuning the in‐plane mode profile at the bound‐leaky boundary, we design metasurface waveguides with uniform modal field distribution in the core region, where the effective index remains invariant with respect to the core width. Our findings encompass not only fully capacitive or inductive scenarios but also complex capacitive‐inductive junctions supporting coupled line waves. Experimental validation through near‐field measurements on a microwave prototype operating in the C band confirms our theoretical predictions. These results hold intriguing potentials for applications in flat optics, sensing, and communications.

## Graphical abstract

The study introduces scale‐invariant metasurface waveguides with uniform modal field distribution and an effective index independent of the core width. By leveraging spatial symmetry and fine‐tuning mode profiles, the design is validated through near‐field measurements in the C band, offering potential applications in flat optics, sensing, and communications.

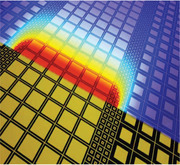

## 1 Introduction

The advent of two‐dimensional (2‐D) materials, both natural [1] (e.g., graphene) and artificial [2, 3] (“metasurfaces”), has revitalized interest in surface‐wave electromagnetics (EM). This resurgence spans across optics, microwave engineering, and antenna design areas where the surface‐wave EM has long been a subject of interest [4]. Despite the increased complexity of the mathematical modeling, it is both feasible and insightful to extend familiar concepts and ideas from conventional (volumetric) configurations to “flatland” scenarios. For instance, inspired by conventional dielectric waveguides and resonators [5], step‐like modulations of surface impedance can be used to create surface waveguides [6, 7] and cavities [8, 9]. These structures replicate the effects of their volumetric counterparts in the plane while maintaining out‐of‐plane confinement. Similarly, more complex refractive devices, such as prisms [10] and lenses [11], can also be designed. Remarkably, even advanced metamaterial‐inspired concepts, such as negative refraction [12], metalenses [13], complex media with extreme parameters [14, 15, 16, 17], photonic crystals [18], and transformation optics [19, 20], can be effectively translated.

Within this framework, it is also possible to identify lower‐dimensional versions of surface waves that are localized both out‐of‐plane and in‐plane. These “line waves” [21, 22] can propagate along suitable planar discontinuities in surface impedance, effectively channeling energy along 1‐D pathways. This analogy has, in turn, enabled the extension of well‐known concepts such as leaky waves [23] to flatland [24], a phenomenon that is similar to 2‐D Cherenkov radiation [25, 26]. Furthermore, 2‐D analogies with more exotic wave phenomena observed in non‐Hermitian [27] and biaxially anisotropic [28] volumetric scenarios have been explored [29, 30, 31, 32, 33].

In this study, we explore the flatland extension of the concept of scale‐invariant waveguiding, a notion recently proposed for conventional dielectric waveguides [34]. We demonstrate that by leveraging spatial symmetry and precisely tuning the in‐plane bound‐leaky mode transition [29], it is possible to design surface waveguides that exhibit a uniform modal field distribution in the central region and are scale‐invariant, that is, the mode propagation constant does not vary regardless of the width of this region. We show that this phenomenon is quite general; it applies not only to fully capacitive or inductive scenarios, where the analogy with dielectric waveguides is straightforward [6], but also to more complex configurations featuring capacitive‐inductive junctions that support coupled line waves. Our theory and numerical results are experimentally validated through near‐field measurements on a microwave prototype operating in the C band. These findings advance the ability to control and tailor the surface‐wave propagation, potentially opening up innovative applications in diverse areas such as polaritonics, sensing, and communications.

## 2 Problem Formulation

**FIGURE 1:**
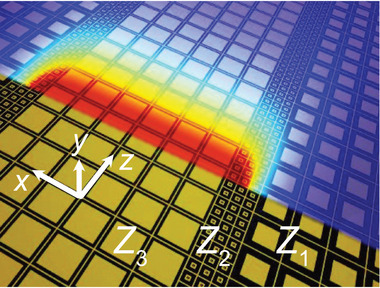
Conceptual illustration of a scale‐invariant surface waveguide.

As conceptually illustrated in Figure 1, our proposed scale‐invariant surface waveguide consists of a metasurface assumed laying in the *x*–*z* plane, with a symmetric modulation of surface impedance along the x‐direction. This design supports a surface‐wave mode that propagates along the z‐direction, decays exponentially in the out‐of‐plane direction (y), and maintains a uniform profile along the x‐direction in the central region. Remarkably, the propagation constant of the mode does not vary, regardless of the width of this central region.

**FIGURE 2:**
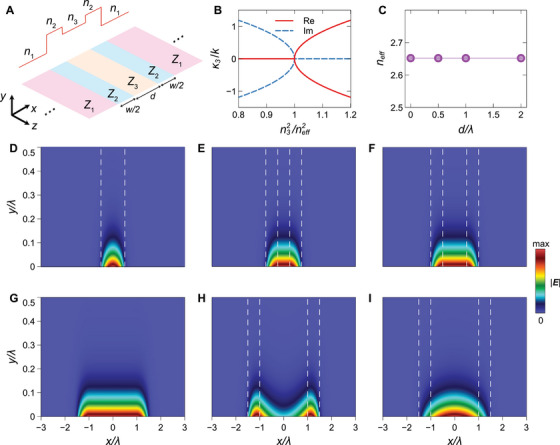
Representative results for the fully inductive configuration. (A) Schematic illustration and effective‐index landscape. (B) Illustration of the bound‐leaky mode transition for a parameter configuration with $Z_{1}=j1.5\eta$, $Z_{2}=j2.5\eta$ and w=λ ($n_{\mathrm{eff}}=2.652$). (C) Numerically computed dispersion diagram under scale‐invariance condition ($Z_{3}=j2.456\eta ,n_{3}=n_{\mathrm{eff}}\;$). (D–G) Numerically computed field maps (|E|) in false‐color scale for d=0,0.5λ,λ,2λ, respectively, under the scale‐invariance condition. (H), (I) Same as above, for d=2λ, and two cases where the scale‐invariance condition is not met, that is, $Z_{3}=j2.41\eta$ ($n_{3}<n_{\mathrm{eff}}$) and $Z_{3}=j2.5\eta$ ($n_{3}>n_{\mathrm{eff}}$), respectively. White‐dashed lines indicate the positions of impedance discontinuities.

We assume that each region is homogeneous and isotropic, thus characterizing the metasurface with an impedance boundary condition:

(1) $$E_{t}=Z\left(x\right)\hat{y}\times {\left.H\right|}_{y=0},$$

where the subscript “*t*” indicates the tangential component, $\hat{y}$ is a y‐directed unit vector, and Z(x) is a symmetric, piecewise‐continuous surface impedance (refer to the schematic in Figure 2A):

(2) $$Z\;\left(x\right)=\left\{\begin{array}{cc} Z_{1}, & \left|x\right|>\left(d+w\right)/2,\\ Z_{2}, & \left|x-d/2\right|<\left(d+w\right)/2,\\ Z_{3}, & \left|x\right|<w. \end{array}\right.\;$$

We also assume an exp(jωt) time‐harmonic dependence and, for now, neglect losses and dispersion, making the surface impedances purely imaginary, that is, $Z_{i}=jX_{i},i=1,2,3$, where the positive and negative signs of the reactances $X_{i}$ correspond to inductive and capacitive sections, respectively. The impact of losses will be addressed in detail later.

It is well‐known that capacitive and inductive metasurfaces support surface waves with transverse electric (TE) and transverse magnetic (TM) polarization, respectively. These waves propagate in the *x*–*z* plane with a propagation constant given by [4]:

(3) $$k_{t}=\left\{\begin{array}{c} k\sqrt{1-{\left(\frac{\eta }{Z}\right)}^{2}},TE,\\ k\sqrt{1-{\left(\frac{Z}{\eta }\right)}^{2}\;},TM, \end{array}\right.\;$$

where k=ω/c=2π/λ and η≈377Ω denote the vacuum wavenumber and intrinsic impedance, respectively, with c and λ representing the corresponding wavespeed and wavelength, respectively. The out‐of‐plane decay is governed by the attenuation constant $\alpha =\sqrt{k_{t}^{2}-k^{2}}\;.$

When all sections are of the same type (i.e., all capacitive or all inductive), the in‐plane response can be viewed as the flatland analog of a dielectric waveguide. In particular, an “effective index” can be defined in the three regions as follows [6]:

(4) $$n_{i}=\frac{k_{\mathrm{ti}}}{k}\;,\;i=1,2,3.$$

This effective index serves a role analogous to the refractive index in dielectric waveguides. Consequently, surface‐waveguiding can be achieved by appropriately designing the surface‐impedance modulation in Equation (2) so that the effective index in the central region ($n_{3}$) is higher than those in the two outer regions. Drawing a parallel with the terminology used for dielectric waveguides, we refer to the central region as the “core” and the outer regions as “cladding”. However, this analogy is not exact. Unlike dielectric waveguides, analytic treatment is generally impossible, and fields are typically hybrid, involving all six (electric and magnetic) components. Even for simple scenarios, such as a single impedance discontinuity, analytical approaches based on Wiener–Hopf [35] or Sommerfeld–Maliuzhinets [36] methods are cumbersome and do not provide a clear physical parameterization. Although a recent approximate analytical method has been proposed [32], its application to multiple interfaces remains complex. Consequently, rigorous analysis often relies on fully numerical approaches, such as the spectral method of moments [37] or finite‐element methods [38].

Nevertheless, this analogy allows for tailoring the surface impedance distribution in Equation (2) to create an effective‐index landscape (see the inset in Figure 2A) that enables scale‐invariant waveguiding. Specifically, inspired by the theory developed for dielectric waveguides [34], the effective index $n_{3}$ of the central region should be chosen to match the effective index of the mode supported by the surface waveguide when the central region is absent (i.e., when d=0).

In the following sections, we investigate, implement, and experimentally validate an inductive configuration. We also conduct a numerical study of dual capacitive configuration, as well as a mixed capacitive/inductive scenario where the dielectric‐waveguide analogy does not apply.

## 3 Results and Discussion

### 3.1 Fully Inductive Configuration

Referring to the schematic in Figure 2A, we examine a fully inductive configuration where the two outer sections have surface impedances $Z_{1}=j1.5\eta$ and $Z_{2}=j2.5\eta$, corresponding to effective indices $n_{1}=1.803$ and $n_{2}=2.695$, respectively. When the central section is absent (i.e., d=0), the structure functions as a conventional surface waveguide with a high‐index core and low‐index cladding, supporting a quasi‐TM fundamental mode with even symmetry [6]. We define as $n_{\mathrm{eff}}=\beta /k$ the effective index of this mode, with β denoting the modal propagation constant along z, which is computed numerically (see Section 6 for details). As shown in Figure S1, this effective index $n_{\mathrm{eff}}\;$ varies with the core width w, and the in‐plane mode profile is characterized by a peak at the center of the core and an evanescent in‐plane decay within the cladding.

We now fix the core width w and introduce a central region with a surface impedance $Z_{3}$ such that its effective index $n_{3}$ precisely matches the modal index $n_{\mathrm{eff}}$ of the unperturbed waveguide for that specific core width. For the chosen parameters in this example (w=λ), the modal effective index is $n_{\mathrm{eff}}=2.652$, and according to Equations (4) and (3) particularized for the TM case, the corresponding surface impedance is $Z_{3}=j2.456\eta .$

To understand the physical implications, Figure 2B illustrates the behavior of the transverse (x‐directed) in‐plane propagation constant in the central region:

(5) $$\kappa_{3}=\sqrt{k_{t3}^{2}-\beta^{2}}=k\sqrt{n_{3}^{2}-n_{\mathrm{eff}}^{2}}$$

near the point where $n_{3}=n_{\mathrm{eff}}\;$. The matching condition distinguishes the bound regime, where $\kappa_{3}$ has purely imaginary values, resulting in an exponentially localized field of the form $\exp(\pm |\kappa_{3}|x)$, from the leaky regime, where $\kappa_{3}$ is purely real, leading to a standing wave formed by counterpropagating terms of the form $\exp(\pm j\kappa_{3}x)$. This transition between bound and leaky modes has been observed in similar contexts involving “flatland” leakage [24], considering only one half of the structure in Figure 2A (i.e., as d→∞). At the matching condition $n_{3}=n_{\mathrm{eff}}\;$, the propagation constant $\kappa_{3}$ becomes zero, resulting in a uniform field across the region with surface impedance $Z_{3}$. When the geometry exhibits spatial mirror symmetry, as in the structure in Figure 2A, this uniform field behavior persists regardless of the size of the central region d, analogous to the behavior observed in dielectric waveguides [34]. As a consequence, we obtain a scale‐invariant mode with a uniform in‐plane distribution in the central region |x|<d and an effective index independent of the width d. This is confirmed by the numerically computed field maps shown in Figure 2D–G (for d=0,0.5λ,λ,2λ, respectively) and the corresponding dispersion diagram in Figure 2C. Interestingly, the uniform field distribution is achieved in a region characterized by a lower effective index, corresponding to a lower surface reactance in this case. The field maps also reveal that the modes remain bound to the surface, with an evanescent decay in the out‐of‐plane direction (along y). For comparison, Figure 2H,I displays field maps for cases where the scale‐invariance condition is not met, resulting in nonuniform field profiles, either peaked at the interfaces ($n_{3}<n_{\mathrm{eff}}$; Figure 2H) or at the center ($n_{3}>n_{\mathrm{eff}}$; Figure 2I).

For completeness, Figures S2 and S3 provide the field components for the modes discussed in Figure 2, illustrating their inherently hybrid nature with all components being generally non‐zero. However, in the central region, sufficiently far from the impedance discontinuities, the modes exhibit a quasi‐TM behavior with dominant components $E_{x},\;E_{y}$, and $H_{z}$.

### 3.2 Realistic Design

The idealized configuration in Figure 2A requires sharp discontinuities in surface impedance, which can pose significant technological challenges. At terahertz or optical frequencies, natural 2‐D (van der Waals) materials can be effectively utilized [1]. For example, as demonstrated in various adaptations of volumetric effects, including waveguiding, graphene platforms can be leveraged through the use of uneven ground planes, dielectric spacers with inhomogeneous permittivity, or by applying gate electric or magnetic fields [19]. Additionally, recent advancements in van der Waals junctions hold significant potential for addressing the challenges associated with sharp interfaces [39, 40].

**FIGURE 3:**
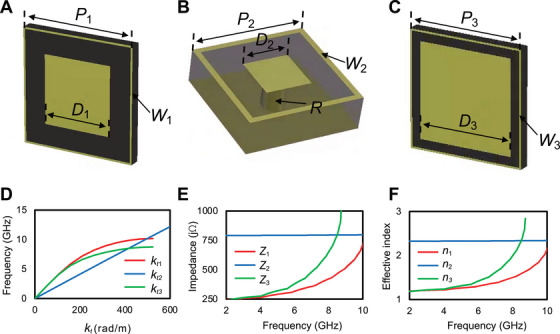
Realistic microwave design. (A–C) Unit‐cells pertaining to surface impedances $Z_{1}$ ($P_{1}=6\;\mathrm{mm},D_{1}=3.6\;\mathrm{mm},W_{1}=0.1\;\mathrm{mm})$, $Z_{2}$ ($P_{2}=2\;\mathrm{mm},D_{2}=0.8\;\mathrm{mm},W_{2}=0.1\;\mathrm{mm},R=0.2\;\mathrm{mm}$), and $Z_{3}$ ($P_{3}=6\;\mathrm{mm},D_{3}=4.9\;\mathrm{mm},W_{3}=0.1\;\mathrm{mm}$), respectively. The yellow areas indicate the metallizations. A 0.6mm‐thick FR4 dielectric substrate is assumed. (D–F) Numerically computed dispersion characteristics, surface impedances, and effective indices, respectively.

In this study, we focus on a more manageable microwave implementation using textured metasurfaces. Figure 3A–C illustrates the unit‐cell designs for achieving the three required surface‐impedance values ($Z_{1},Z_{2},Z_{3}$). We utilize finite‐integration simulations [41] to extract the effective constitutive parameters of these unit cells (see the Section 6 for more details). Specifically, Figure 3D presents the numerically computed dispersion characteristics ($k_{t}$ vs. frequency), from which we derive the corresponding surface impedance and effective index using Equations (3) and (4), as shown in Figure 3E,F, respectively.

As can be observed, the constitutive parameters for unit cells of types 1 and 3 exhibit strong dispersion, while those for the type 2 unit cell are only mildly dispersive. Assuming w=12mm, numerical simulations indicate that the scale‐invariance condition $n_{3}=n_{\mathrm{eff}}\;$ is approximately satisfied around 7.8 GHz. Due to the noted dispersion, this condition can only be achieved within a narrow bandwidth.

### 3.3 Experimental Validation

**FIGURE 4:**
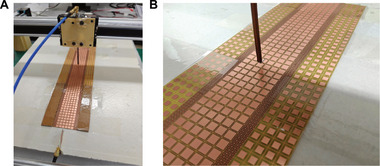
Experimental validation. (A) Measurement setup. (B) Detailed view of the prototype and the measuring probe (mostly polarized along the z‐direction).

**FIGURE 5:**
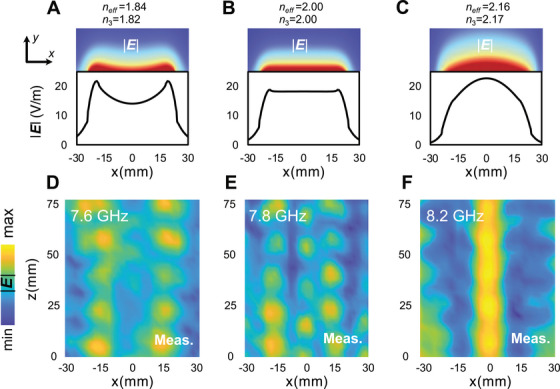
Comparison between simulated and measured results. (A–C) Numerically computed modal field distributions (out‐of‐plane maps and field profiles at y=0mm) pertaining to the designed prototype at three frequencies: below (7.6 GHz; $Z_{1}=j1.05\eta ,Z_{2}=j2.11\eta ,Z_{3}=j1.52\eta$), at (7.8 GHz; $Z_{1}=j1.12\eta ,Z_{2}=j2.11\eta ,Z_{3}=j1.73\eta$), and above (8.2 GHz; $Z_{1}=j1.18\eta ,Z_{2}=j2.11\eta ,Z_{3}=j1.93\eta$) the scale‐invariance condition, respectively. (D–F) Corresponding measurements (in‐plane maps at y=2mm).

Figure 4 displays the fabricated prototype along with the near‐field scanning system used to measure the field distributions (see the Section 6 for more details). Figure 5 presents a comparison between simulations and measurements. Specifically, Figure 5A–C shows the simulated modal field distributions, including out‐of‐plane (*x*–*y*) maps and field profiles at y=0mm, at three frequencies: below (7.6 GHz), at (7.8 GHz), and above (8.2 GHz) the scale‐invariance condition, respectively. The results are consistent with the observations in Figure 2.

Figure 5D–F presents the corresponding measurements in terms of in‐plane (*x*–*z*) field maps. The measurements confirm our theoretical predictions, demonstrating the expected distinct behaviors under the three conditions: $n_{3}<n_{\mathrm{eff}}$ (Figure 5D), $n_{3}=n_{\mathrm{eff}}\;$(Figure 5E), and $n_{3}>n_{\mathrm{eff}}$ (Figure 5F). Notably, the field distribution in the scale‐invariance case exhibits an extended behavior in the central region, albeit with some oscillations. This imperfect response can be attributed to the strong dispersion of the metasurfaces, which makes the matching condition difficult to achieve exactly, as well as to the unavoidable truncations of the outermost sections (of surface impedance $Z_{1}$), which generate parasitic reflections and a standing‐wave‐type pattern. Moreover, the metasurfaces consist of a periodic arrangement of metal patches, with each unit cell containing regions not covered by metal. This local inhomogeneity leads to deviations from an ideal field distribution. Finally, reflections at the interface between $Z_{3}$ and $Z_{2}$ significantly affect the field distribution in the central region, particularly due to the limited number of unit cells with surface impedance $Z_{3}$ along the x‐direction. Despite these imperfections, the results demonstrate a proof‐of‐principle validation.

### 3.4 Alternative Configurations

An obvious alternative to the fully inductive configuration considered so far is a fully capacitive one. Through simple duality considerations, it can be shown that the effective‐index landscape remains unchanged under the impedance transformation *Z* $\to \eta^{2}/Z$. The response of the dual (fully capacitive) configuration, corresponding to the scenario in Figure 2, is illustrated in Figures S4–S6. The behavior is analogous, except for the quasi‐TE nature of the fields, with the electric and magnetic fields interchanged.

A less straightforward alternative, explored in the Supporting Information, involves a combination of capacitive and inductive sections. In this case, the dielectric‐waveguide analogy does not apply. Instead, this scenario can be seen as a low‐dimensional equivalence of an insulator‐metal‐insulator heterostructure [42], where line waves propagating at the capacitive‐inductive interfaces [21, 22] play a role similar to that of surface plasmon polaritons at metal‐dielectric interfaces. This capacitive‐inductive scenario was also studied in connection with flatland leakage [24], and the corresponding in‐plane bound‐leaky transition identifies the scale‐invariance condition. Some representative results are shown in Figure S7–S9. We observe all the typical features of scale invariance, although the field amplitude in the central region may be significantly smaller than those at the capacitive‐inductive interfaces due to the strong field‐enhancement effect of line waves [21, 22].

In principle, the scale‐invariance condition can be achieved in a wide range of surface waveguides that exhibit spatial symmetry.

## 4 Some Remarks on Losses and Bandwidth

In the examples discussed so far, we have assumed ideal, lossless (i.e., purely reactive) structures. In the Supporting Information, we extend this analysis to include the effects of losses, starting with an idealized surface‐impedance model that incorporates a resistive component in the materials. To simplify this initial study, we neglect dispersion and assume a uniform loss tangent across all materials. Figure S10A shows that, for an inductive scenario, increasing the loss tangent keeps the real part of the effective index nearly constant (with variations only in the fourth decimal place), while the imaginary part (representing attenuation along the *z*‐axis) increases in magnitude. Additionally, Figure S10B shows that, for a fixed loss tangent, both the real and imaginary parts of the effective index are largely unaffected by the size of the central region d.

To provide a more realistic assessment, we also investigate the impact of dielectric losses in our microwave implementation, estimating an attenuation constant of α=13.94 Np/m at the operational frequency of 7.8 GHz (see Figure S11 and the related discussion in the Supporting Information).

Regarding bandwidth, as previously discussed in the context of the dielectric‐waveguide scenario (see Section 8 in the Supporting Information of ref. [34]), the scale‐invariance condition is strictly valid at a single frequency, making it inherently a narrow‐band effect. However, due to the relatively flat behavior of the dispersion relationship in Equation (5) near the critical point, enhanced field overlap in the central region can be observed across a broader band, provided that material dispersion is minimal. In our microwave implementation, bandwidth limitations are further affected by the inherent dispersion of the periodic structures we utilize [43]. It is important to note that our design serves as a simple proof of principle and was not optimized for bandwidth stability; thus, more frequency‐stable implementations could be developed in principle. Additionally, at higher frequencies, the use of natural 2‐D materials [1], such as graphene, could help mitigate dispersion and expand the bandwidth range.

## 5 Conclusions

In summary, we have introduced the concept of scale‐invariant surface waveguides, which are formed by suitably tailored, symmetric planar junctions of metasurfaces. These waveguides feature a uniform field distribution in the central region and exhibit an effective modal index that remains independent of the width of this region. This concept, inspired by a dielectric‐waveguide analogy [34], is also closely tied to the recently introduced notion of flatland leakage [24].

We have investigated, both theoretically and numerically, the emergence of this phenomenon across various configurations, including fully inductive, fully capacitive, and mixed setups, highlighting similarities and differences compared to the volumetric counterpart. Finally, for proof‐of‐principle validation, we have designed, fabricated and characterized a microwave prototype. One of the intriguing features of the proposed concept is the ability to concentrate the field in regions with a relatively low effective index (i.e., low surface inductance or high surface capacitance). Similar to the dielectric‐waveguide analogy [34], it is theoretically possible to operate with small contrast, with the operational bandwidth being primarily limited by dispersion.

Overall, these results offer enhanced capabilities and greater flexibility in controlling surface‐wave propagation, unlocking new opportunities across various research domains. For instance, in the emerging field of polaritonics [44, 45], this approach could broaden the range of useful material properties and manipulation techniques. In sensing applications, enhanced field uniformity can improve the effectiveness and control of light‐matter interactions, leading to more accurate measurements. A uniform electromagnetic field can favor consistent interactions, boosting sensitivity and signal‐to‐noise ratio, especially for low‐concentration biomolecules. It may also allow precise control over resonance excitation and may enhance reproducibility by minimizing local field strength variations.

Additionally, the microwave surface impedance of superconductors can be dynamically tuned by temperature [46, 47] or an external optical fields [48], making the development of superconducting scale‐invariant detectors a foreseeable possibility [49].

Moreover, in wireless communications, investigating the out‐of‐plane dimension—an aspect absent in the dielectric‐waveguide analogy—presents a promising research opportunity. By introducing periodic modulation of surface impedance along the propagation direction, it may be possible to couple a scale‐invariant guided mode with radiative modes in the surrounding medium. This strategy could pave the way for the development of innovative metasurface antennas [50] and advanced surface‐wave‐based smart radio environments [51]. Within this context, the scale‐invariance property can be leveraged to enhance system performance by facilitating effective coupling between surface‐wave modes and narrow beams.

## 6 Methods

### 6.1 Numerical Modeling

All numerical simulations of the idealized structures were performed using the finite‐element‐based commercial software package COMSOL Multiphysics [38]. Specifically, we employed the “Mode Analysis” study within the RF Module, and considered a 2‐D semi‐circular computational domain terminated by a perfectly matched layer (PML) with a thickness of 0.5λ. Impedance boundary conditions at y=0 were implemented via an equivalent surface current density with components: $J_{x}=E_{x}\;/Z_{i},J_{y}=0,J_{z}=E_{z}\;/Z_{i}$, where i=1,2,3. The domain was adaptively meshed with an element size with element size ≤0.01λ (and ≤0.002λ nearby the impedance surfaces). The MUMPS direct solver was used with default settings. For the realistic design, the unit cells shown in Figure 3 were simulated using the eigenmode solver in CST Studio Suite [41]. The dielectric substrate (FR4) was modeled with a relative permittivity of $\varepsilon_{r}=4.4$ and a loss tangent of 0.02, while the metallizations were represented as copper (electrical conductivity $\sigma \;=5.8\cdot 10^{7}\;S/m$). A parallelepiped computational domain was used, featuring an air layer of thickness 10 mm above the unit cell, an ideal electric conductor boundary 10 mm from the unit cell along the y direction, and Floquet‐type boundary conditions in the *x*–*z* plane. The in‐plane propagation constant $k_{t}$ was extracted from the resulting band diagram, assuming propagation along the x‐direction [52].

### 6.2 Prototype Fabrication and Characterization

The prototype was fabricated using standard printed‐circuit‐board (PCB) technology on a commercial FR4 dielectric substrate with a thickness of 0.6mm. Referring to the schematic in Figure 2A, the dimensions are d=36mm and w=12mm. The outermost sections, which are ideally of infinite extent, were truncated to a width of 24mm at each end. For experimental characterization, a near‐field scanning system was employed to measure field distributions in the *x*–*z* plane. The setup includes an Agilent N5230A vector network analyzer (VNA), with one port connected to a Vivaldi antenna for excitation, and the other port connected to an electric probe. The probe, positioned 2mm above the metasurface, samples the local electric fields, and its movement is controlled by an Arduino Uno.

Further details on the Vivaldi antenna and additional numerical results on the angular sensitivity of the surface‐wave excitation are provided in the Supporting Information (see Figures S12–S14).

## Conflicts of Interest

The authors declare no conflict of interest.

## Supporting information




**Supporting Information file 1**: exp270108‐sup‐0001‐SuppMat.docx

## Data Availability

The data that support the findings of this study are available from the corresponding authors upon reasonable request.
