# Supplementary material for: Scale‐Invariant Waveguiding in Flatland
Source: Exploration (Beijing). 2026 Jan 28;6(1):70108. doi: 10.1002/exp2.70108 (PMC12970178; doi:10.1002/exp2.70108)
Supplement: Supplementary file 1 — Supporting Information file 1: exp270108‐sup‐0001‐SuppMat.docx [file EXP2-6-70108-s001.docx]

Supporting Information

Scale-Invariant Waveguiding in Flatland

Zhixia Xu, Shuo Bao, Massimo Moccia, Giuseppe Castaldi, Tie Jun Cui*
and Vincenzo Galdi*

This document contains additional numerical results and details on the experimental setup. Newly introduced figures are labeled with the prefix “S”; all others pertain to the main text.

**Additional Results**

Figure S1 illustrates the dispersion characteristics and modal field distribution for a conventional surface waveguide.

Figure S2 and Figure S3 show the field components for the modes discussed in Figure 2.

Figures S4, Figure S5, and Figure S6 illustrate the response of the dual (fully capacitive) configuration with respect to the case shown in Figure 2. As can be observed, the behavior is analogous, except for the quasi-TE nature of the fields, with the electric and magnetic fields interchanged.

Figure S7A shows an alternative configuration combining capacitive and inductive sections. For a representative example, we assume $Z_{1}=-j5.6\eta$ (capacitive) and $Z_{2}=j\eta$ (inductive).

Without the central region $d=0$, this configuration supports a fundamental even mode originating from the line-wave coupling. Assuming $w=0.6\lambda$, the effective index of this mode, computed numerically, is $n_{eff}=2.059$. This capacitive-inductive scenario was also studied in connection with flatland leakage,^[S1]^ and the corresponding in-plane bound-leaky transition identifies the scale-invariance condition. Accordingly, we assume an inductive center section with a surface impedance $Z_{3}$ selected to satisfy the matching condition $n_{3}=n_{eff}$ (see Figure S7B). From the results shown in Figure S7C-G, we observe the typical hallmarks of scale invariance, which disappear when this condition is not met (see Figures S7H,I). However, compared to the fully inductive (Figure 2) and fully capacitive (Figure S4) scenarios, the field amplitude in the central region is significantly smaller than at the capacitive-inductive interfaces due to the strong field-enhancement effect characteristic of line waves.^[S2,S3]^ This is more evident in the modal profiles shown in Figure S8 and Figure S9, where a semi-log scale is used for better visibility.

**
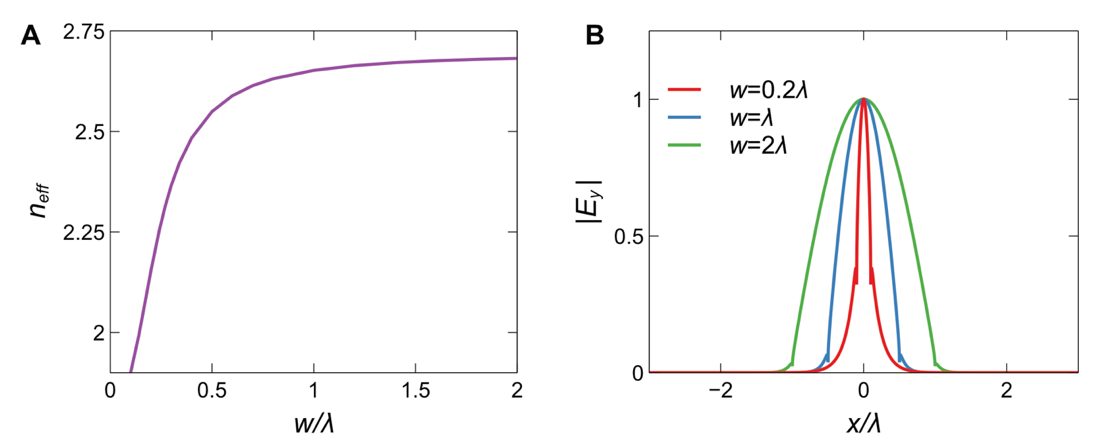
**

**FIGURE S1.** Conventional surface waveguide. **(**A) Numerically computed dispersion diagram of a conventional inductive surface waveguide with $d=0$, $Z_{1}=j1.5\eta$ and $Z_{2}=j2.5\eta$. (B) Numerically computed modal field profiles for selected values of the core width (for simplicity, only the $y$-component of the electric field is displayed).


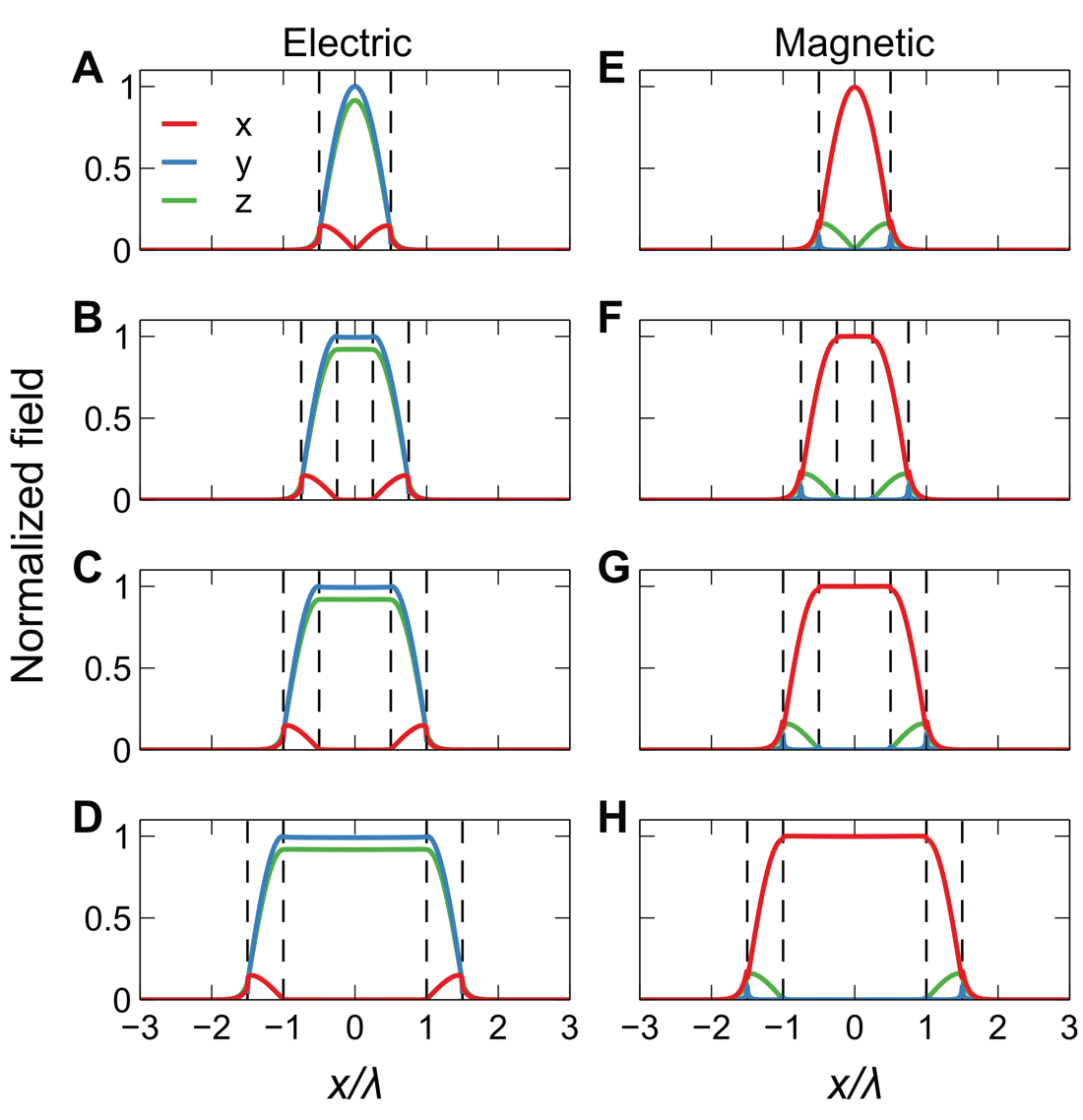


**FIGURE S2.** Parameters as in Figure 2. (A), (B), (C), (D) Numerically computed electric field components for the scale-invariant modes with $d=0,0.5\lambda,\lambda,2\lambda$, respectively, evaluated at $y=0.001\lambda$. (E), (F), (G), (H) Corresponding magnetic field components. Black-dashed lines indicate the positions of impedance discontinuities.


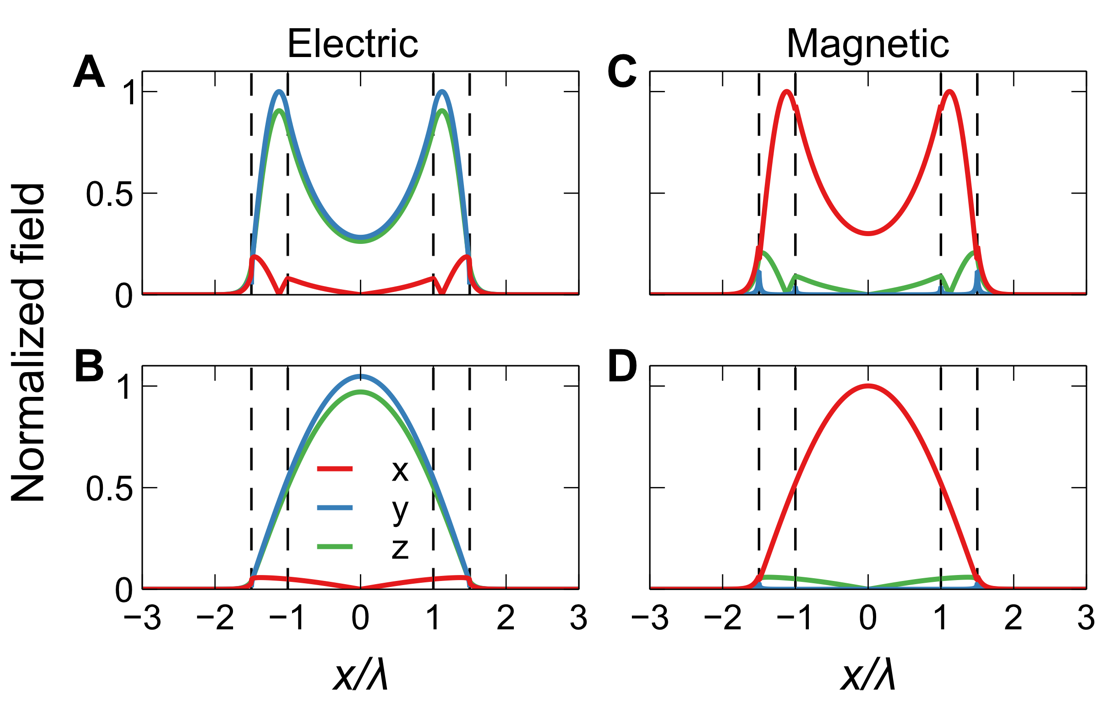


**FIGURE S3.** Parameters as in Figure 2. (A), (B) Numerically computed electric field components for $d=2\lambda$, and two cases where the scale-invariance condition is not met, i.e., $Z_{3}=j2.41\eta$ ($n_{3}<n_{eff}$) and $Z_{3}=j2.5\eta$ ($n_{3}>n_{eff}$), respectively, evaluated at $y=0.001\lambda$. (C), (D) Corresponding magnetic field components. Black-dashed lines indicate the positions of impedance discontinuities.


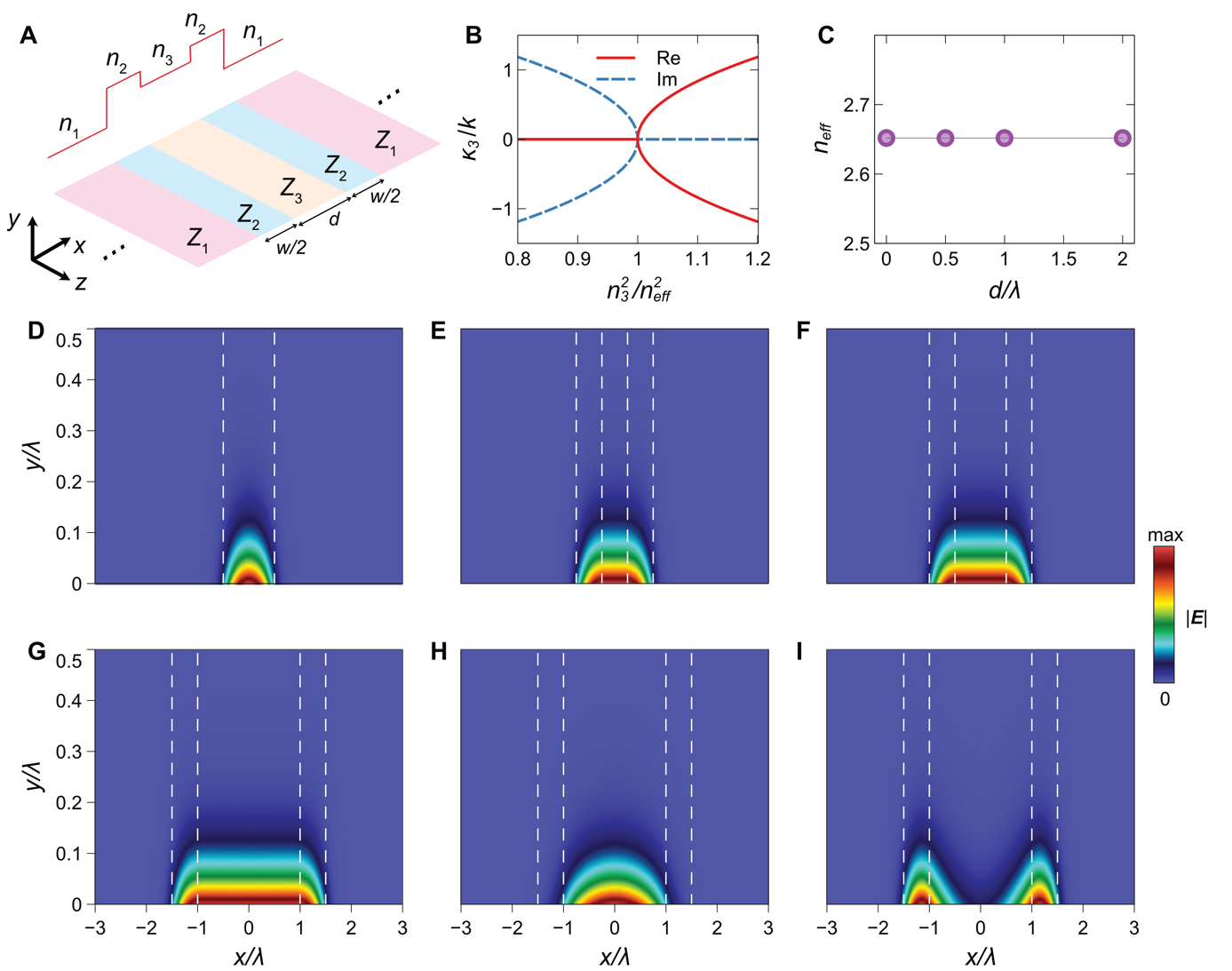


**FIGURE S4.** Representative results for dual (fully capacitive) configuration. (A) Schematic illustration and effective-index landscape. (B) Illustration of the bound-leaky transition for a parameter configuration with $Z_{1}=-j0.667\eta$, $Z_{2}=-j0.4\eta$ and $w=\lambda$ ($n_{eff}=2.652$). (C) Numerically computed dispersion diagram under scale-invariance condition ($Z_{3}=-j0.407\eta,n_{3}=n_{eff}$). (D), (E), (F), (G) Numerically computed field maps ($\left| \boldsymbol{E} \right|$) in false-color scale for $d=0,0.5\lambda,\lambda,2\lambda$, respectively, under scale-invariance condition. (H), (I) Same as above, for $d=2\lambda$, and two cases where the scale-invariance condition is not met, i.e., $Z_{3}=-j0.38\eta$ ($n_{3}>n_{eff}$) and $Z_{3}=-j0.42\eta$ ($n_{3}<n_{eff}$), respectively. White-dashed lines indicate the positions of impedance discontinuities.

**
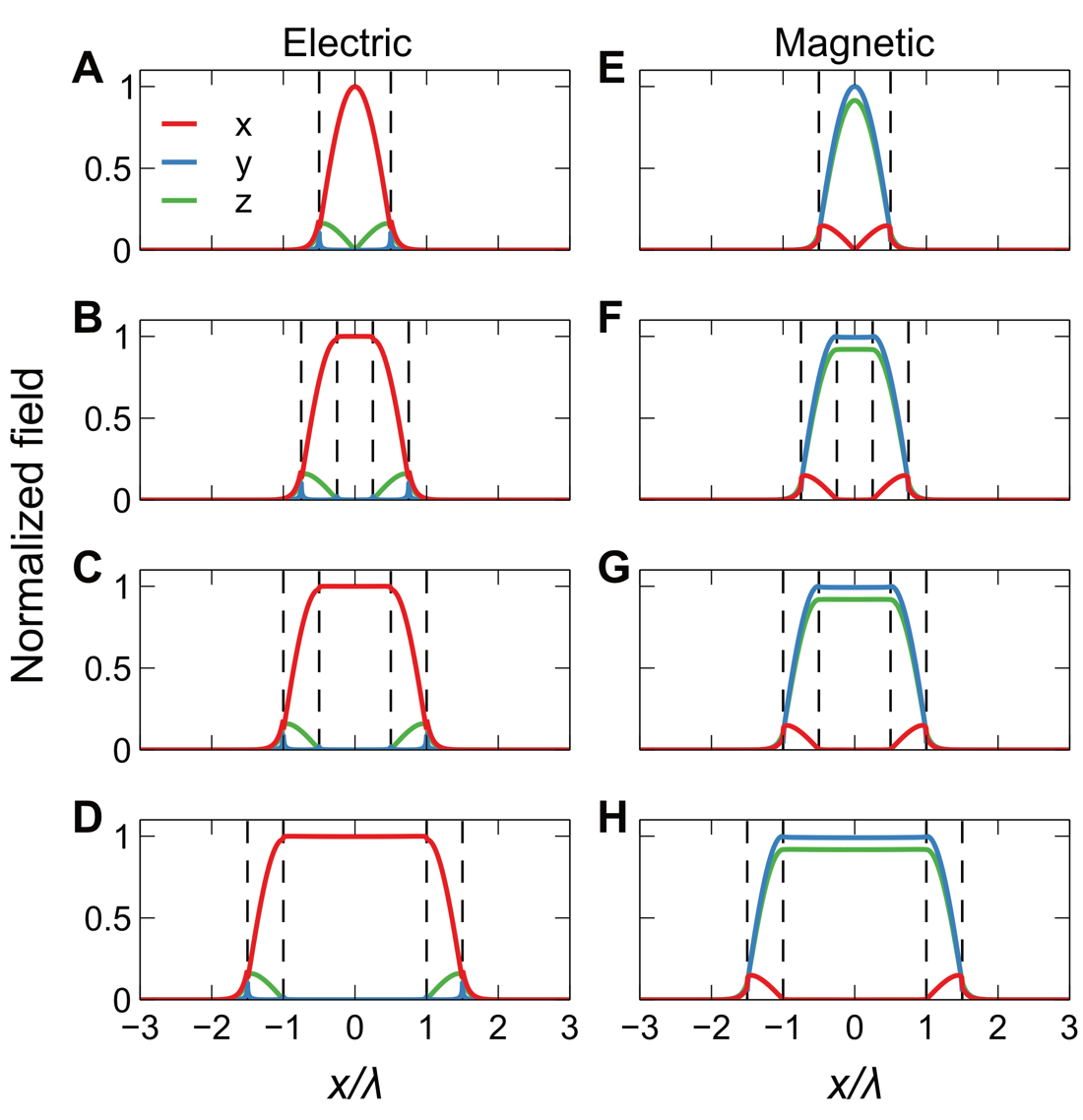
**

**FIGURE S5.** Parameters as in Figure S4. (A), (B), (C), (D) Numerically computed electric field components for the scale-invariant modes with $d=0,0.5\lambda,\lambda,2\lambda$, respectively, evaluated at $y=0.001\lambda$. (E), (F), (G), (H) Corresponding magnetic field components. Black-dashed lines indicate the positions of impedance discontinuities.


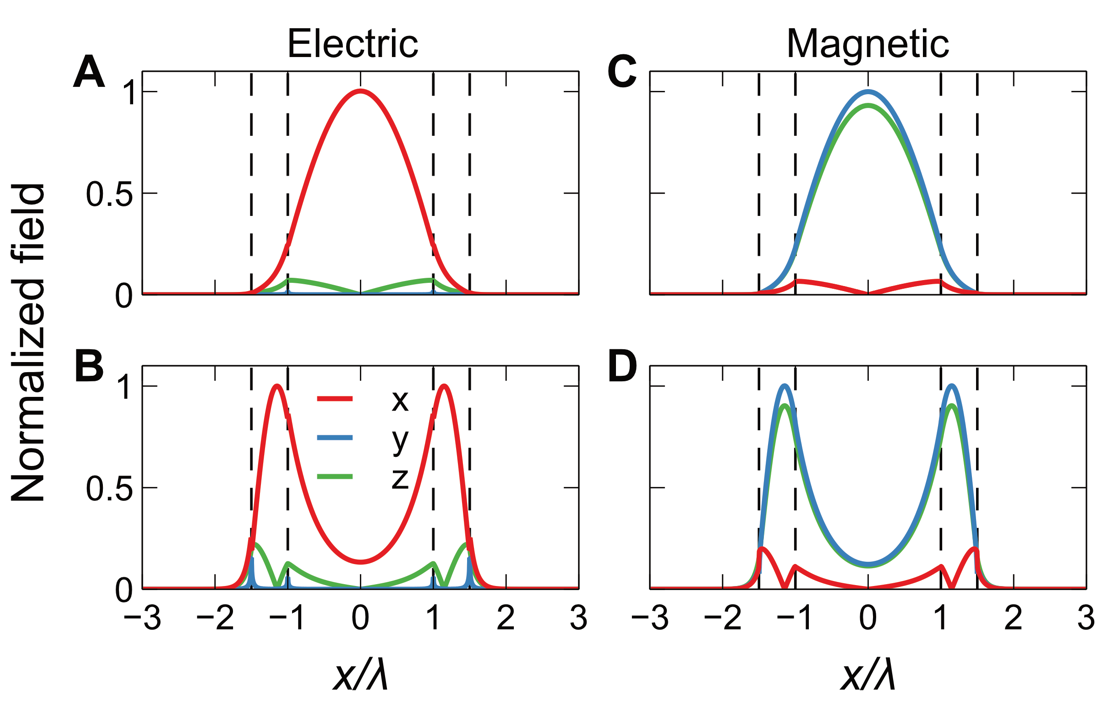


**FIGURE S6.** Parameters as in Figure S4. (A), (B) Numerically computed electric field components for $d=2\lambda$, and two cases where the scale-invariance condition is not met, i.e., i.e., $Z_{3}=-j0.4\eta$ ($n_{3}>n_{eff}$) and $Z_{3}=-j0.415\eta$ ($n_{3}<n_{eff}$), respectively, evaluated at $y=0.001\lambda$. (C), (D) Corresponding magnetic field components. Black-dashed lines indicate the positions of impedance discontinuities.

**
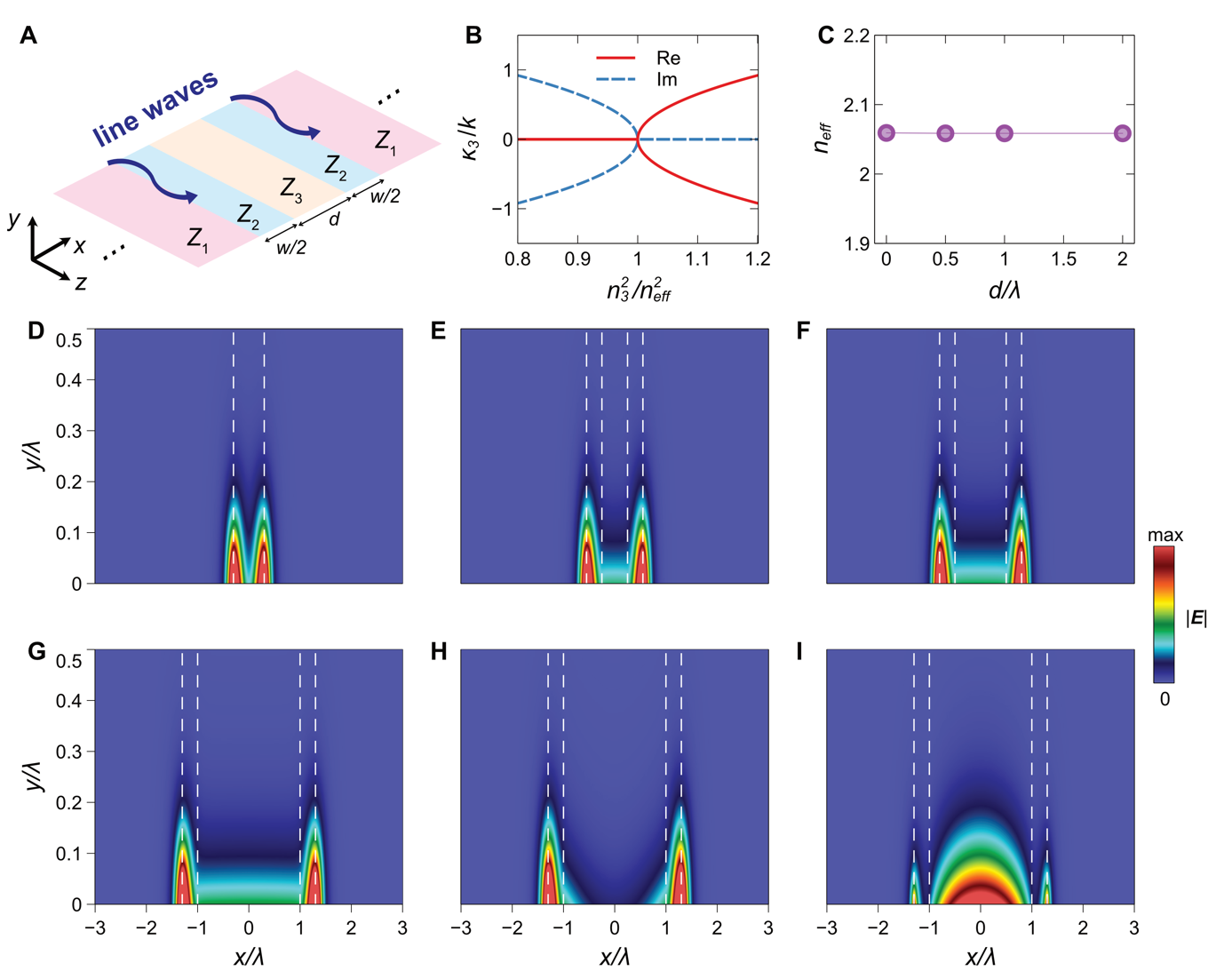
**

**FIGURE S7.** Representative results for capacitive-inductive configuration. (A) Schematic illustration and effective-index landscape. (B) Illustration of the bound-leaky transition for a parameter configuration with $Z_{1}=-j5.6\eta$, $Z_{2}=j\eta$ and $w=0.6\lambda$ ($n_{eff}=2.059$). (C) Numerically computed dispersion diagram under scale-invariance condition ($Z_{3}=j1.8\eta,n_{3}=n_{eff}$). (D), (E), (F), (G) Numerically computed field maps ($\left| \boldsymbol{E} \right|$) in false-color scale for $d=0,0.5\lambda,\lambda,2\lambda$, respectively, under scale-invariance condition. (H), (I) Same as above, for $d=2\lambda$, and two cases where the scale-invariance condition is not met, i.e., $Z_{3}=j1.77\eta$ ($n_{3}<n_{eff}$) and $Z_{3}=j1.83\eta$ ($n_{3}>n_{eff}$), respectively. White-dashed lines indicate the positions of impedance discontinuities.


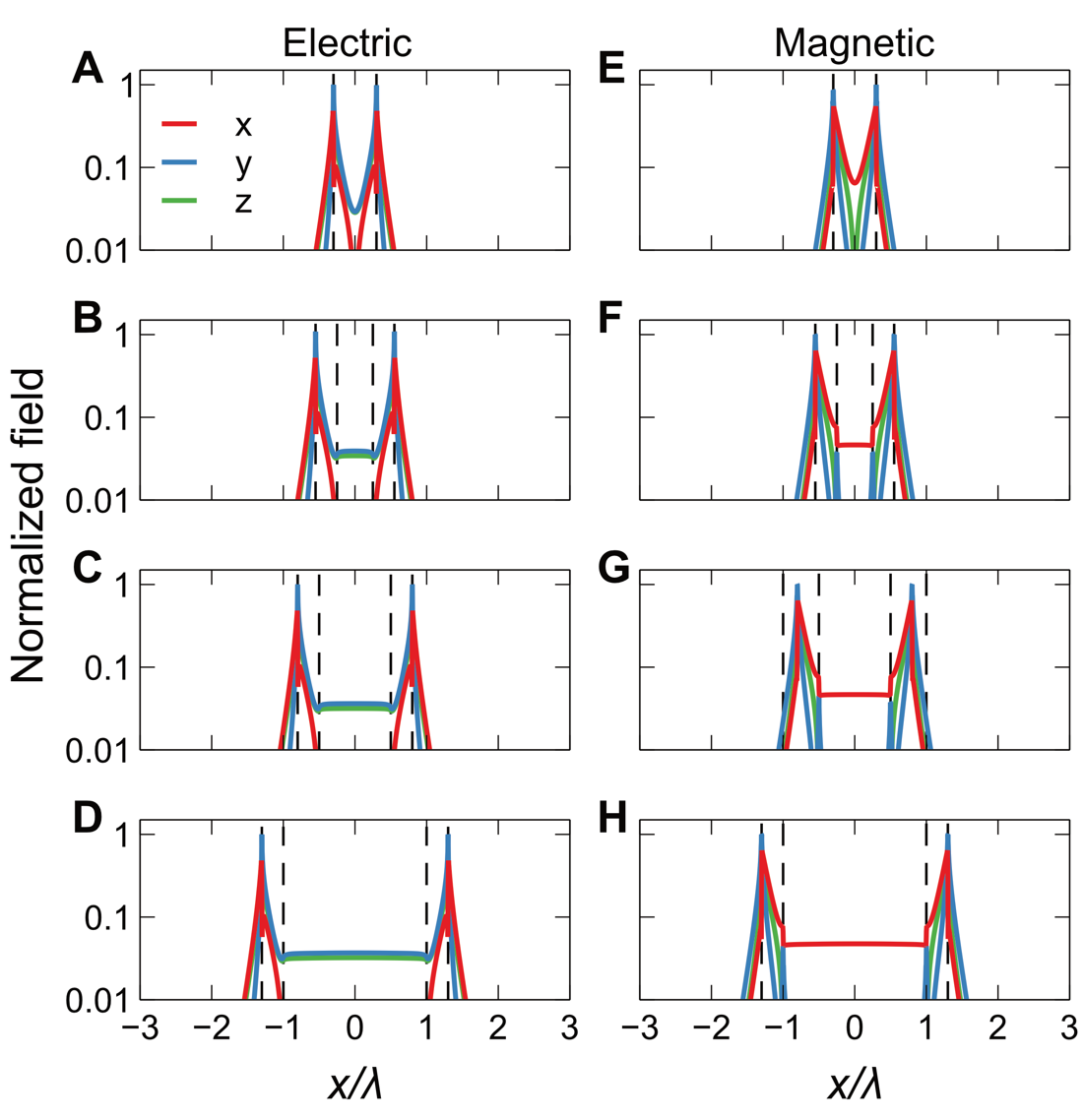


**FIGURE S8.** Parameters as in Figure S7. (A), (B), (C), (D) Numerically computed electric field components for the scale-invariant modes with $d=0,0.5\lambda,\lambda,2\lambda$, respectively, evaluated at $y=0.001\lambda$. (E), (F), (G), (H) Corresponding magnetic field components. Black-dashed lines indicate the positions of impedance discontinuities. Note the semi-log scale.

**
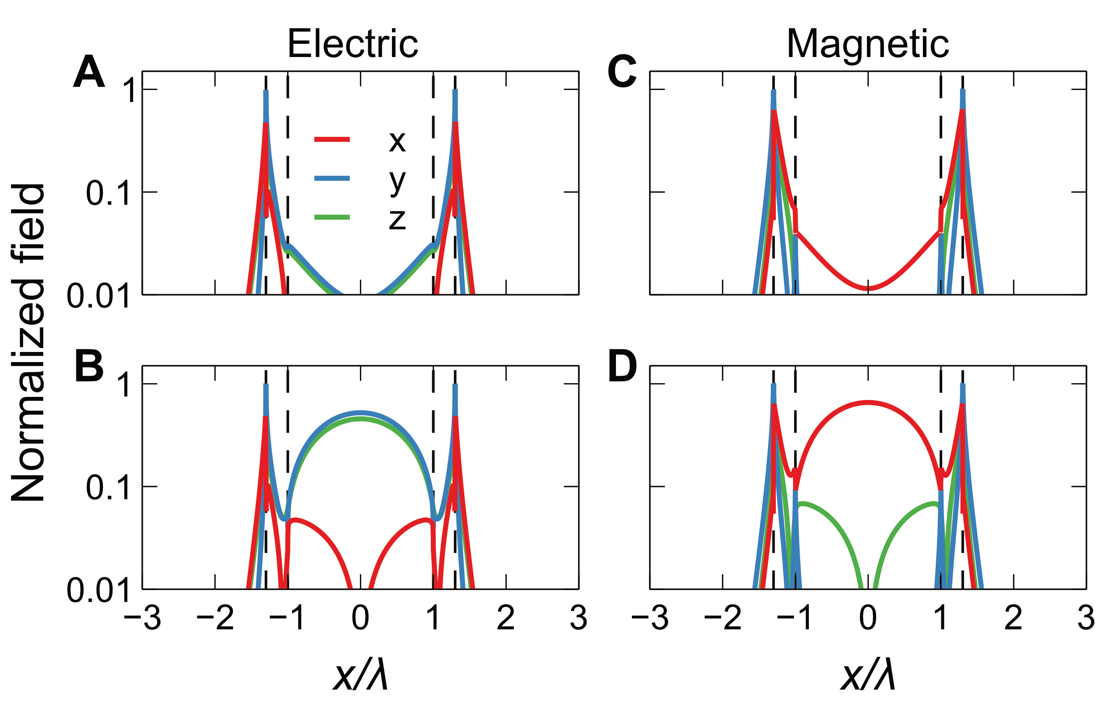
**

**FIGURE S9.** Parameters as in Figure S7. (A), (B) Numerically computed electric field components for $d=2\lambda$, and two cases where the scale-invariance condition is not met, i.e., $Z_{3}=j1.77\eta$ ($n_{3}<n_{eff}$) and $Z_{3}=j1.83\eta$ ($n_{3}>n_{eff}$), respectively, evaluated at $y=0.001\lambda$. (C), (D) Corresponding magnetic field components. Black-dashed lines indicate the positions of impedance discontinuities. Note the semi-log scale.

**Effect of Losses**

To examine the impact of losses, we start with the ideal fully inductive setup in Figure 2A and introduce a resistive component in the surface impedances. We parameterize these losses using a simple, nondispersive model with a loss tangent, defined as $\tan\delta=R/X$, applied uniformly to the three materials. This addition makes the modal index $n_{eff}$ complex. For the same surface reactances as in Figure 2 and a fixed width $d$ of the central region, Figure S10A shows that the loss tangent affects the real and imaginary parts of the modal index. While the real part remains nearly constant (varying only in the fourth decimal place), the imaginary part (which represents attenuation along the *z*-axis) grows in absolute value. As shown in Figure S10B, for a fixed loss tangent, the complex modal index remains largely independent of the central region's width. Thus, similar to the case of dielectric waveguides,^[S4]^ the scale-invariance property holds despite the presence of losses, albeit with an expected attenuation effect.

As a more realistic example, we consider the microwave design in Figures 3–6, accounting for the intrinsic loss of the dielectric substrate while ignoring metallic losses. For the FR4 substrate, we assume a loss tangent of 0.02, resulting in a complex relative permittivity $\varepsilon=\varepsilon^{'}-j\varepsilon^{''}=4.4-j0.088$. We conduct a driven-mode simulation using an array of point dipole sources at approximately 7.8 GHz to estimate the decay rate $\alpha$ of the scale-invariant transmission mode. Figure S11A–D shows the perpendicular electric field distribution $E_{y}$ across various cross-sections. Additionally, we plot ${|E}_{y}|$ along the center line (3 mm above the metasurface) to minimize surface interference from the unit cell. Through exponential curve fitting $\left| E_{y} \right|=\left| E_{max} \right|e^{-\alpha z}$, we determine $\alpha$ as shown in Figure S11F, beginning at $z\geq25$ mm to exclude direct interference from the sources. The resulting decay rate is $\alpha= 13.94$ Np/m.

We further validate this result with an eigenmode analysis of the supercell in Figure S11E. By sweeping the phase constant $\beta$, we obtain the complex eigenfrequency $\Omega\left( \beta\right)=\omega_{r}+j\omega_{i}$, which allows us to deduce the complex propagation constant $\gamma(\omega_{0})=\alpha(\omega_{0})+j\beta(\omega_{0})$ to assess the decay rate.^[S5]^ Using the approximation $\omega_{0}\approx\omega_{r}$, with $\omega_{r}\gg\omega_{i}$, we find $\alpha\approx{\omega_{i}}/{v_{g}}$,^[S6]^ where $v_{g}$ is the group velocity, resulting in an estimated $\alpha\approx10.3$ Np/m. Both results approximate the decay rate, as scattering boundaries affect it to some degree. The scale-invariant mode can propagate about 0.1 m, with the amplitude decaying to $1/e$ of its original value at 7.8 GHz.


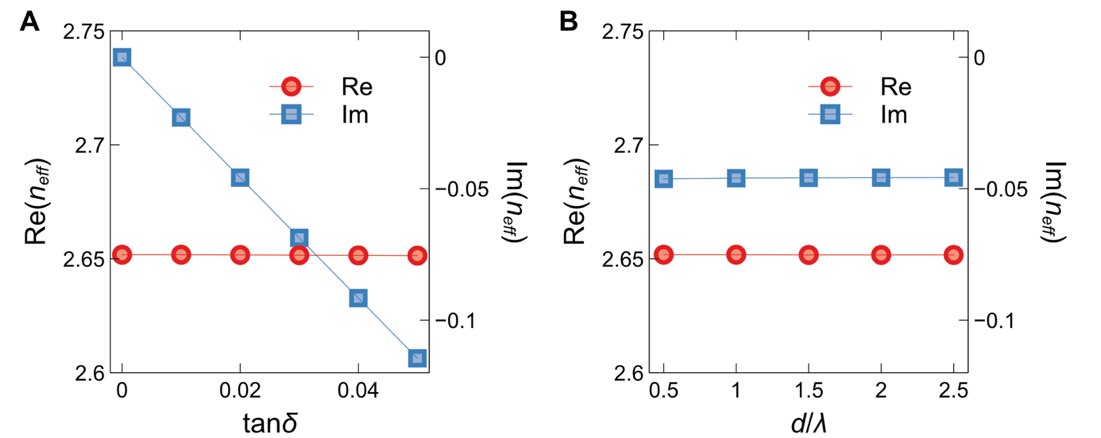


**FIGURE S10.** Effect of losses: Idealized configuration (see Figure 2). (A) Real and imaginary part of mode index as a function of loss tangent, for the parameter configuration in Figure 2, and $d=2\lambda.$ The three surface impedances are parameterized as $Z_{1}=j1.5 (1+j\tan\delta)\eta$, $Z_{2}=j2.5(1+j\tan\delta)\eta$, and $Z_{3}=j2.456 (1+j\tan\delta)\eta$. (B) Real and imaginary part of mode index for fixed loss tangent ($\tan\delta=0.02$), as a function of central-region width.


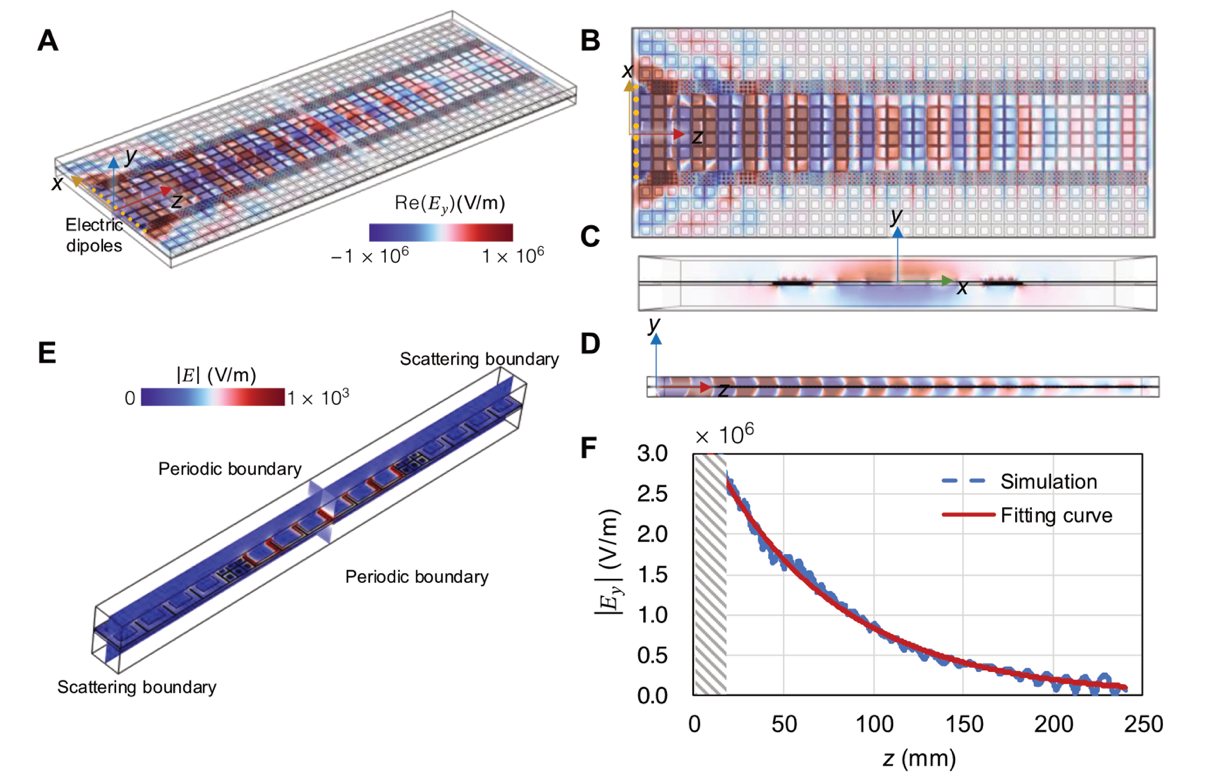


**FIGURE S11.** Effect of losses: Realistic configuration (see Figures 3-5). (A) 3D model excited by ideal point electric dipoles along the *y*-axis. (B), (C), (D) Field distributions [$\mathrm{Re}(E_{y})$] on the $x-z$, $x-y$, and $y-z$ planes, respectively. (E) Eigenmode analysis of the supercell. (F) Simulated and numerically fitted $E_{y}$ magnitude at 7.8 GHz.

**Details on the Vivaldi Antenna**

The Vivaldi antenna utilized in the experimental setup is based on a low-impedance slotted-line configuration, as shown in Figure S12A. It is fabricated on a $30\times30\times1$ mm^3^ RO4003C dielectric substrate with patterned copper cladding on both surfaces. The slotted-line design features precision-etched slots in the copper layers, with closely spaced metallized vias along the slot edges, electrically connecting the upper and lower cladding layers. Since both the via radius and the via-to-slot-edge distance are significantly smaller than the operational wavelength across the working band, the via array effectively acts as a continuous metallic boundary, ensuring well-defined electromagnetic confinement for the slot-line structure.

Figure S12B presents a photograph of the fabricated antenna, while Figure S12C and Figure S12D illustrate its reflection coefficient ($\left| S_{11} \right|$, simulated and measured) and simulated far-field radiation pattern, respectively. The results demonstrate that the antenna operates efficiently from 5 GHz to 20 GHz, exhibiting a highly directional beam.

**Additional Results on Angular Sensitivity of Surface-Wave Excitation**

To further validate the robust excitation of the desired surface-wave modes on the metasurface by the Vivaldi antenna, we systematically analyze its angle-insensitive excitation characteristics. As illustrated in Figure S13, we compare two distinct metasurface configurations: a conventional uniform high-impedance metasurface and our proposed scale-invariant metasurface waveguide, under both normal ($\theta=0^{\circ}$) and oblique ($\theta={20}^{\circ}$) in-plane incidence.

For the uniform metasurface (Figure S13A,B), surface waves are successfully excited in both cases. However, their propagation exhibits significant spatial dispersion, lacking well-defined waveguiding trajectories. In contrast, the scale-invariant metasurface waveguide (Figure S13C,D) preserves clear transmission paths under both excitation conditions. The consistent wavefront evolution patterns at both incidence angles confirm the guided wave mode’s incident-angle immunity, highlighting the proposed structure’s superior mode confinement capability.

The observed background field leakage primarily stems from the finite-size nature of the excitation source. As shown in Figure S10, reducing the source dimensions to an idealized point configuration significantly enhances the excitation efficiency and purity of the scale-invariant mode. This sensitivity to source dimensions necessitates careful consideration in experimental implementations. While COMSOL Multiphysics^[S7]^ allows for the use of idealized point sources, CST Studio Suite^[S8]^ requires practical antenna structures—such as the implemented Vivaldi antenna or an electric probe—to achieve detectable power levels. These differences between simulation platforms should be carefully considered when translating theoretical models into physically realizable systems.

We have further developed an effective surface impedance model in COMSOL Multiphysics, excited by a phased array of ideal point sources, to validate the angle-insensitive excitation of the waveguide system. The array comprises 10 ideal point sources, spaced at $\lambda/4$ intervals, with a tunable feeding phase difference ($\Delta\phi$) of 0°, 10°, 20°, or 30°. Even varying the array’s radiation angle, the waveguide mode is consistently and selectively excited, confirming the angle-insensitivity of the scale-invariant waveguide mode, as illustrated in Figure S14.


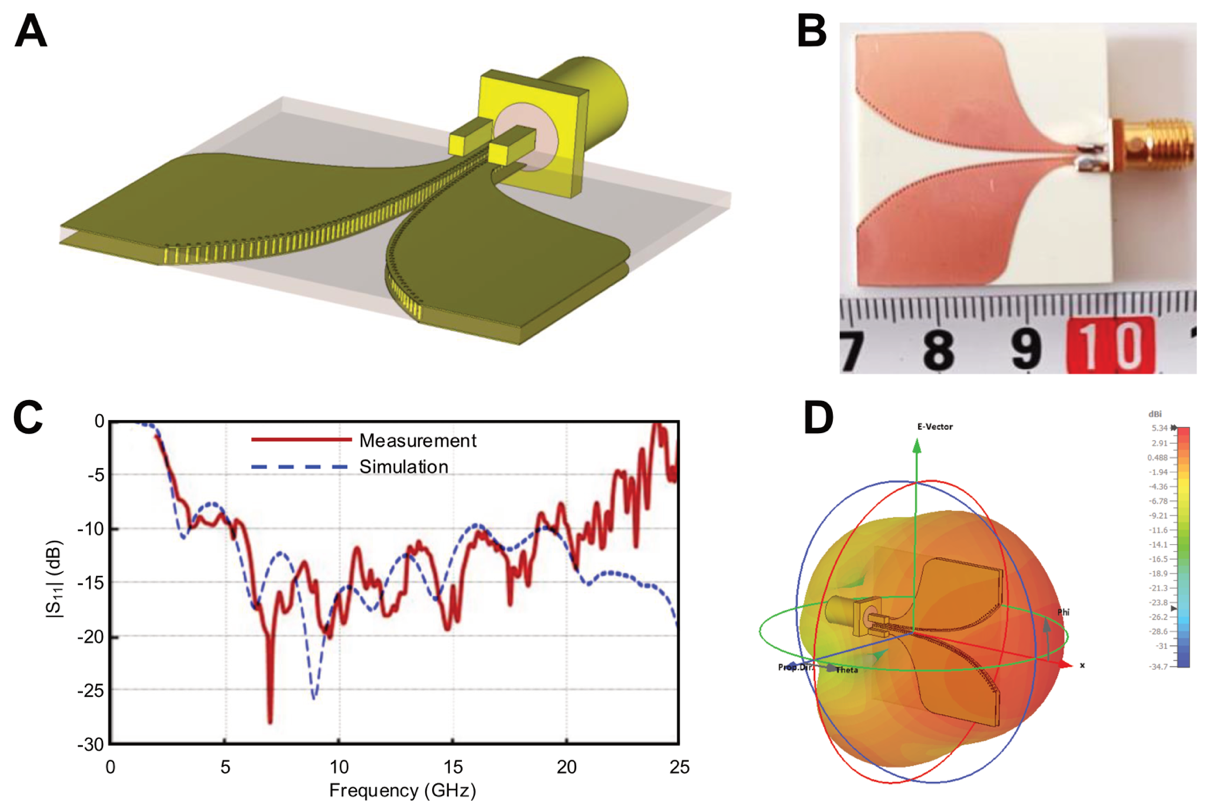


**FIGURE S12.** Overview of the Vivaldi antenna utilized as a source. (A) Antenna configuration. (B) Photograph of the fabricated antenna. (C) Simulated and measured $|S_{11}|$. (D) Simulated far-field radiation pattern at 7.5 GHz.


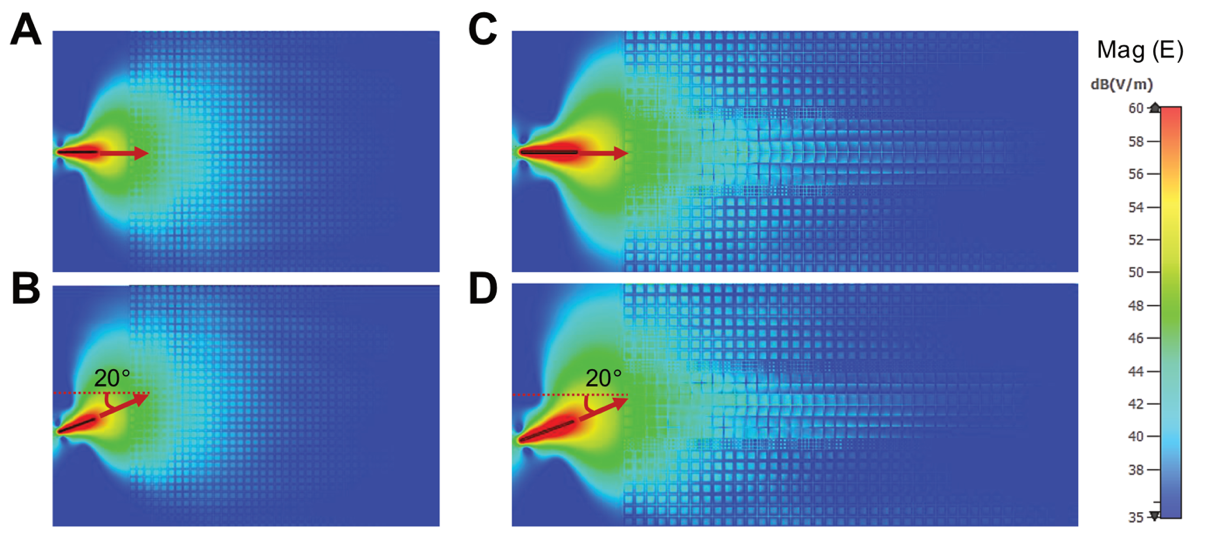


**FIGURE S13.** Vivaldi antenna as a source, simulated via CST Studio Suite. (A), (B) In-plane field maps for normal ($\theta=0^{\circ}$) and oblique ($\theta={20}^{\circ}$) incidence, respectively, on a uniform metasurface with surface impedance Z_3_ (as in Figure 3C). (C), (D) Same as panels (A), (B), respectively, but for a scale-invariant metasurface waveguide, as in Figure 3 and Figure 4.


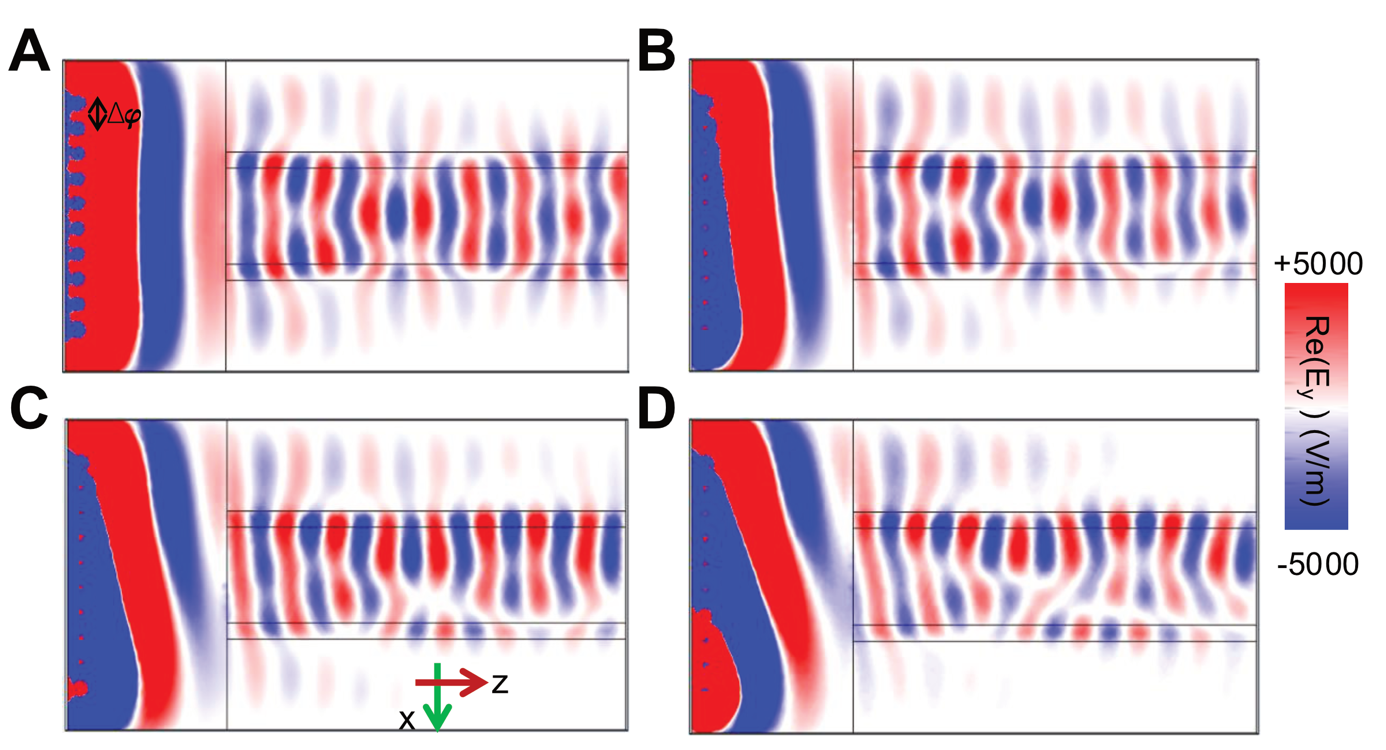


**Figure S14.** In-plane field maps, computed via COMSOL Multiphysics, for a scale-invariant surface waveguide excited by a phased array of ideal point sources generating different incidence angles. (A) $\Delta\varphi=0$. (B) $\Delta\varphi=10^{\circ}$. (C)$\Delta\varphi=20$°. (D) $\Delta\varphi=30^{\circ}$. The simulated homogeneous model features ideal impedance surfaces with $Z_{1}=j1.12\eta, Z_{2}=j2.11\eta, Z_{3}=j1.73\eta$, as in Figure 5B.

**References**

[S1] M. Moccia, G. Castaldi, A. Alù, V. Galdi, *Adv. Opt. Mater.* **2024**, *12*, 2203121.

[S2] S. A. R. Horsley, I. R. Hooper, *J. Phys. D: Appl. Phys.* **2014**, *47*, 435103.

[S3] D. J. Bisharat, D. F. Sievenpiper, *Phys. Rev. Lett.* **2017**, *119*, 106802.

[S4] J. R. Rodrigues, U. D. Dave, A. Mohanty, X. Ji, I. Datta, S. Chaitanya, E. Shim, R. Gutierrez-Jauregui, V. R. Almeida, A. Asenjo-Garcia, M. Lipson, *Nat. Commun.* **2023**, *14*, 6675.

[S5] J. G. N. Rahmeier, V. Tiukuvaara, S. Gupta, *IEEE Transactions on Antennas and Propagation* **2021**, *69*, 4644.

[S6] E. Sauer, J. P. Vasco, S. Hughes, *Phys. Rev. Res.* **2020**, *2*, 043109.

[S7] COMSOL Multiphysics® v. 5.1, https://www.comsol.com.

[S8] CST Studio Suite 3D EM simulation and analysis software, https://www.3ds.com/products-services/simulia/products/cst-studio-suite.
